# Ryanodine Receptors Selectively Interact with L Type Calcium Channels in Mouse Taste Cells

**DOI:** 10.1371/journal.pone.0068174

**Published:** 2013-06-27

**Authors:** Michelle R. Rebello, Amanda B. Maliphol, Kathryn F. Medler

**Affiliations:** Department of Biological Sciences, University at Buffalo, The State University of New York, Buffalo, New York, United States of America; University of Tokyo, Japan

## Abstract

**Introduction:**

We reported that ryanodine receptors are expressed in two different types of mammalian peripheral taste receptor cells: Type II and Type III cells. Type II cells lack voltage-gated calcium channels (VGCCs) and chemical synapses. In these cells, ryanodine receptors contribute to the taste-evoked calcium signals that are initiated by opening inositol trisphosphate receptors located on internal calcium stores. In Type III cells that do have VGCCs and chemical synapses, ryanodine receptors contribute to the depolarization-dependent calcium influx.

**Methodology/Principal Findings:**

The goal of this study was to establish if there was selectivity in the type of VGCC that is associated with the ryanodine receptor in the Type III taste cells or if the ryanodine receptor opens irrespective of the calcium channels involved. We also wished to determine if the ryanodine receptors and VGCCs require a physical linkage to interact or are simply functionally associated with each other. Using calcium imaging and pharmacological inhibitors, we found that ryanodine receptors are selectively associated with L type VGCCs but likely not through a physical linkage.

**Conclusions/Significance:**

Taste cells are able to undergo calcium induced calcium release through ryanodine receptors to increase the initial calcium influx signal and provide a larger calcium response than would otherwise occur when L type channels are activated in Type III taste cells.

## Introduction

There are functionally distinct populations of mammalian taste receptor cells that use different mechanisms to generate evoked signals. Type I taste cells are thought to act primarily as support cells, while Type II cells detect bitter, sweet and umami stimuli by activating a G-protein dependent signaling pathway to cause calcium release from internal stores [1]–[3]. Type II cells do not express voltage-gated calcium channels (VGCCs) and do not have conventional chemical synapses [4], [5] but instead express hemichannels and release ATP as a neurotransmitter [6]–[8]. Type III taste cells have calcium influx signals through VGCCs [5], [9] and can detect sour stimuli [10]. This population of taste cells has conventional chemical synapses and releases neurotransmitters such as serotonin and norepinephrine [11], [12].

We recently determined that ryanodine receptors (RyRs) are found in a subset of both Type II and Type III taste receptor cells [13]. Their functional roles vary by cell type and appear to be controlled by the presence or absence of VGCCs. In Type II taste cells which do not express VGCCs, ryanodine receptors contribute to the calcium signal that depends on release from stores as a result of activating the G-protein dependent signaling pathway. However, in taste cells that express VGCCs, RyRs exclusively contribute to the calcium influx signal and do not add to the evoked calcium release signal [13]. These data indicate the potential existence of a relationship between the RyRs and VGCCs in taste cells that is well established in muscle and to a lesser extent in neurons [14]–[18]. However, this connection in taste cells has not been described.

These functional effects also indicate that ryanodine receptors are expressed in some Type II taste cells as well as some Type III cells. In this earlier study, we used immunocytochemistry and RT-PCR analysis to determine that ryanodine receptors, specifically ryanodine receptor type 1, are expressed in about 30% of Type II taste cells but we did not measure its expression in Type III cells [13]. We also did not determine if RyRs are specifically associated with a certain VGCC isoform or if these receptors contribute to the calcium signal when any VGCC is activated. The goal of this study was to better define the nature of the interaction between VGCCs and RyRs. Using calcium imaging and pharmacological blockers, we determined that some calcium influx signals are shaped by a functional, but unlikely a physical, interaction that is specifically between RyRs and L type calcium channels.

## Materials and Methods

### Taste Receptor Cell Isolation

Taste receptor cells were harvested from the taste papillae of transgenic mice expressing GFP under the GAD67 promoter (GAD67–GFP) obtained from Jackson Labs (cat#007677, Bar Harbor, ME, USA). Both sexes of mice were used and mice ranged in age from 1 to 6 months (n = 50 mice total). Mice were sacrificed with carbon dioxide and cervical dislocation. Tongues were removed from animals and injected under the lingual epithelium with an enzymatic solution containing 0.7 mg collagenase B (Roche, Indianapolis, IN, USA), 3 mg dispase II (Roche) and 1 mg trypsin inhibitor (Sigma, St Louis, MO, USA) per milliliter of Tyrode’s solution (140 mM NaCl, 5 mM KCl, 1 mM MgCl_2_, 3 mM CaCl_2_, 10 mM HEPES, 10 mM glucose and 1 mM pyruvic acid; pH 7.4). Tongues were incubated in oxygenated Tyrode’s solution for 20 min before the epithelium was peeled from the connective and muscular tissue. The peeled epithelium was incubated for 30 min in Ca^2+^/Mg^2+^-free Tyrode’s solution (140 mM NaCl, 5 mM KCl, 10 mM HEPES, 2 mM BAPTA, 10 mM glucose and 1 mM pyruvic acid; pH 7.4) before taste cells were removed with a capillary pipette and plated onto glass cover slips coated with Cell-Tak (BD Bioscience, Bedford, MA). Taste cells were viable for several hours. All animal studies were approved by the University at Buffalo Animal Care and Use Committee under protocol number #BIO010174N.

### Calcium Imaging

Isolated taste receptor cells were plated into a laminar flow chamber and loaded at room temperature for 40 min with 2 µM fura 2-AM (Molecular Probes, Invitrogen) containing the nonionic dispersing agent Pluronic F-127 (Molecular Probes, Invitrogen). Loaded cells were visualized using an Olympus IX71 microscope with a 40X oil-immersion lens and images were captured using a SensiCam QE camera (Cooke, Romulus, MI, USA) and Workbench 5.2 (Indec Biosystems, Santa Clara, CA, USA). Excitation wavelengths of 340 and 380 nm were used with an emission wavelength of 510 nm. Fluorescence values were calibrated using the Fura-2 Calcium Imaging Calibration kit (Molecular Probes, Invitrogen). The effective dissociation constant, *K*_d_, was 255 nM, and calcium concentrations were determined using the formula outlined by Grynkiewicz [19]. GAD67-GFP expressing cells were identified using an excitation wavelength of 490 nM and an emission wavelength of 525 nM.

### Immunocytochemistry

For these experiments, we used transgenic mice expressing GFP under the GAD67 promoter (GAD67–GFP). Forty micron sections were cut and washed in phosphate-buffered saline (PBS) three times for 10 min each at room temperature. Sections were initially blocked for 2 h at room temperature in blocking solution (0.3% Triton X-100, 1% normal goat serum and 1% bovine serum albumin in 0.1 m PBS), and then incubated with rabbit anti-RyR1 (Millipore, Billerica, MA, USA, 1∶500 in blocking solution) for 2 h at room temperature, and then left overnight in the primary antibody at 4°C. Following overnight incubation, sections were washed three times for 10 min in PBS and incubated with a goat anti-rabbit rhodamine-tagged antibody (1∶250) (Jackson ImmunoResearch Laboratories, West Grove, PA, USA) at room temperature in the dark for 2 h. Following incubation with secondary antibodies, sections were washed and then mounted on slides using Fluoromount-G (Southern Biotechnology Associates, Birmingham, AL, USA).

Sections were viewed with a three-channel laser scanning confocal microscope with krypton–argon lasers on a Nikon Diaphot 200. Sequential scanning techniques were used. Images were captured with a cooled CCD camera, and Axiovision software was used for data acquisition. Images were processed using Adobe Photoshop CS software adjusting only brightness and contrast.

### Data Analysis

An evoked response was defined as measurable if the increase in fluorescence was more than two standard deviations above baseline. Experimental results were plotted as calcium concentrations and analyzed using OriginPro software (OriginLab Corp., Northampton, MA, USA). Because the response profiles can be quite variable, values were normalized to the initial evoked signal for each cell. Calcium increases were calculated as [(peak − baseline)/baseline] × 100 and were reported as percent increases over baseline. To determine the percent inhibition of the evoked calcium influx by the different inhibitors, calcium responses in the presence of the inhibitor were calculated as a percentage of the previous control depolarization-evoked calcium response using the following formula: [(control response-inhibited response)/control response]*100. This allowed us to measure how much the inhibitor affected the evoked response. Percent of inhibition is reported for each condition tested. Since evoked calcium signals can vary even within a particular cell, the effect of the inhibitor was based on the evoked calcium signal immediately prior to applying the inhibitor. Statistical comparisons were made using either Student’s *t*-test or one-way ANOVAs with a Bonferroni’s *post-hoc* analysis. For all analyses, a significance level of *P*<0.05 was used and standard errors of the mean were reported.

### Solutions

All solutions were bath applied using a gravity flow perfusion system (Automate Scientific, San Francisco, CA, USA) and laminar flow perfusion chambers (RC-25F; Warner Scientific, Hamden, CT, USA). The following solutions were used during experiments: HiK (Tyrode’s solution with 50 mM NaCl replaced by 50 mM KCl or 30 mM NaCl replaced by 30 mM KCl), nimodipine (10 µM), ω-conotoxin GVIA (800 nM), ω-agatoxin IVA (300 nM), nickel chloride (200 µM), and ryanodine (20 µM). All chemicals were purchased from Tocris unless otherwise noted. Calcium-free external solution was comprised of Tyrode’s solution without calcium and magnesium added (140 mM NaCl, 5 mM KCl, 10 mM HEPES, 10 mM glucose and 1 mM pyruvic acid; pH 7.4).

## Results

**Figure 1:**
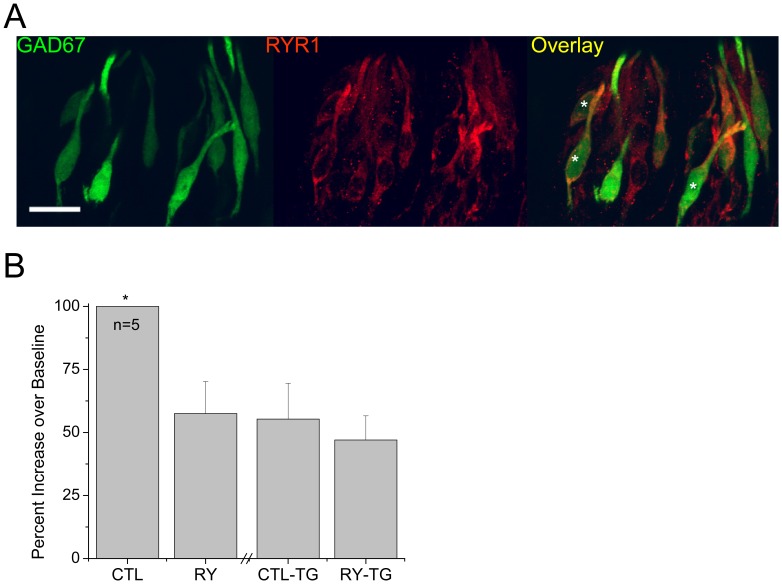
Many GAD67-GFP taste cells express ryanodine receptors. A) Immunocytochemical analysis reveals partial overlap of GAD67-GFP expression (green) and RyR1 immunoreactivity (red). Left panel shows GAD67-GFP expression, middle panel shows RyR1 immunoreactivity and right panel shows the overlay. Representative cells with co-localization are starred (*). Scale bar = 20 µM. B) Analysis using a one-way ANOVA with Bonferroni’s post-hoc analysis found that ryanodine significantly inhibited the depolarization induced calcium influx (n = 4, *p = 0.013). After thapsigargin application, ryanodine had no significant effect on the calcium influx.

The protein glutamic acid decarboxylase 67 (GAD67), which synthesizes the inhibitory neurotransmitter GABA is recognized as a marker for Type III taste cells which express VGCCs and have conventional chemical synapses [9], [20], [21]. Therefore, we used GAD67-GFP transgenic mice to identify a subset of Type III taste cells that express VGCCs and determined if these cells also express ryanodine receptors. We tested for the expression of RyR1 in the GAD67-GFP expressing taste cells and found partial co-localization of RyR1 with GFP expression, confirming that some GAD67-GFP taste cells also express ryanodine receptors (Figure 1A).

For our subsequent experiments, taste cells were determined to express VGCCs by their responsiveness to cell depolarization with 50 mM KCl and were identified as Type III taste cells [5]. GFP expression was assessed in these taste cells to determine if they expressed GAD67. We analyzed both GFP expressing taste cells with VGCCs and GFP negative taste cells with VGCCs to determine if there were any significant differences in the role of ryanodine receptors between these two groups. We also performed control experiments to determine the selectivity of the ryanodine effects on the evoked calcium signals (Figure 1B). If ryanodine application is inhibiting ryanodine receptors that are only located on internal calcium stores, then depletion of these stores will prevent any further contribution to the evoked calcium influx. We analyzed Type III taste cells that have ryanodine receptors contributing to the evoked calcium signal before and after the thapsigargin application. We found that after thapsigargin application, the evoked calcium signal was reduced to levels comparable to the 50 mM KCl +20 µM ryanodine evoked signal prior to thapsigargin. Ryanodine also failed to have any further effect on the evoked signal. We concluded that 20 µM ryanodine is selectively inhibiting ryanodine receptors on internal calcium stores and that when these stores are depleted, there is no calcium release through the ryanodine receptors to contribute to the depolarization evoked calcium signal resulting in a smaller evoked calcium signal.

### VGCCs and Ryanodine Receptors

In an earlier study, we found that ryanodine receptors contribute to the calcium-influx response through voltage-gated channels in mouse taste cells [13]. Our findings suggested an interaction between ryanodine receptors and voltage-gated calcium channels but the nature of this relationship was unknown. We did not know if there was selectivity in the interaction between the ryanodine receptor and particular voltage-gated calcium channel types and we did not know if this interaction was a physical or just a functional one. To assess the types of voltage-gated calcium channels that could be associated with ryanodine receptors, we used pharmacological blockers for specific VGCCs to identify their presence in the Type III taste cells that also express ryanodine receptors.

Isolated taste cells were depolarized with 50 mM potassium chloride (hi K, 10 s), which causes VGCCs to open and generates a calcium influx. After calcium levels recovered to baseline, hi K was applied again in the presence of a high concentration of ryanodine (20 µM) which blocks ryanodine receptors [22]. If the depolarization induced calcium signal was inhibited by blocking the ryanodine receptors, we concluded that RyRs were being functionally expressed and normally contribute to the depolarization induced calcium signal. After recovery, the cell was subsequently depolarized and specific pharmacological blockers for individual VGCCs were applied to determine which channel isoforms were functionally expressed in the taste cell. In order to minimize signal rundown in these experiments, we did not test all of the calcium channel blockers on a particular taste cell but instead grouped and compared responses from multiple taste cells.

### Assessing Potential Interactions between Ryanodine Receptors and VGCCs

We wished to determine if ryanodine receptors preferentially associate with a particular VGCC isoform since we and others [9], [23] have found that multiple VGCCs can be expressed in a given taste cell. To determine if the ryanodine receptors were associating with a particular type of VGCC, we tested two predictions. First, we predicted that if ryanodine receptors were not functionally associated with a particular calcium channel, then on inhibition of both ryanodine receptors and that particular VGCC isoform, the subsequent inhibition of the depolarization evoked calcium signal would be greater. This increased inhibition would occur because the portion of the calcium signal that was normally due to the particular VGCC isoform being inhibited and the portion of the calcium signal that was due to the opening the ryanodine receptor on the internal calcium stores were each making independent contributions to the depolarization induced calcium signal. Therefore, our second prediction was that if the ryanodine receptor was functionally associated with a particular VGCC isoform, then inhibiting both that particular VGCC isoform and the ryanodine receptor should not change the amount of inhibition caused by inhibiting the VGCC alone. That is because the contribution to the calcium signal from that particular VGCC isoform would also be dependent on the opening of the ryanodine receptor, so blocking the VGCC isoform would also inhibit the ryanodine receptor effects.

**Figure 2:**
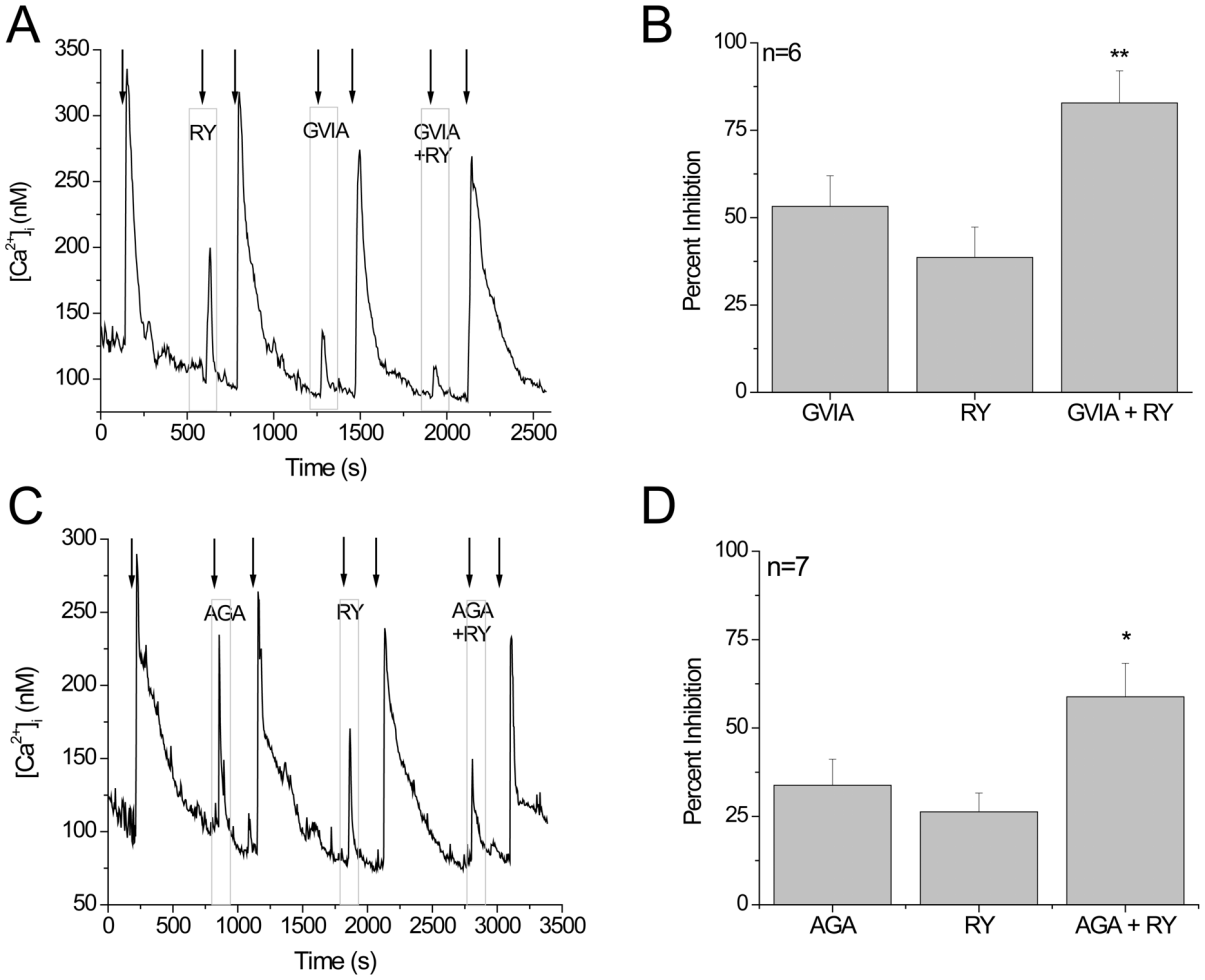
RyRs do not contribute to calcium signal due to N and P/Q type calcium channels. A) Representative trace showing inhibition of the HiK response by ryanodine (20 µM), conotoxin GVIA (800 nM) and conotoxin GVIA+ ryanodine. B) Analysis using a one-way ANOVA with Bonferroni’s post-hoc analysis of the inhibition of the peak amplitude for each condition shows enhanced inhibition of the HiK response during co-application of conotoxin GVIA and ryanodine (n = 6, **p = 0.009) compared to the inhibition due to either N type channels or ryanodine receptors alone. C) Representative trace showing inhibition of the HiK response by agatoxin (300 nM), ryanodine (20 µM) and agatoxin+ryanodine. D) Co-application of agatoxin and ryanodine significantly increases inhibition of the peak amplitude for the calcium-influx response (n = 7, *p = 0.018) compared to the level of inhibition due to blocking either P/Q type channels or ryanodine receptors.

We applied ω-conotoxin GVIA (800 nM) to preferentially block N type calcium channels (Ca_V_2.2) which have previously been identified in mouse taste cells using RT-PCR analysis of isolated taste buds [9]. We found that N type calcium channels were present in some taste cells that express ryanodine receptors and that inhibition of the calcium influx was significantly enhanced on co-application of the blockers (Figure 2A, 2B). Based on these data, we concluded that ryanodine receptors and N type calcium channels are acting independently of each other and there is no functional interaction between them.

We next used ω-agatoxin IVA (300 nM) to selectively inhibit P/Q type calcium channels (Ca_V_2.1) in order to determine if ryanodine receptors and P/Q type channels are functionally interacting. We found that while P/Q type channels were sometimes expressed in the same taste cells that express ryanodine receptors, they did not appear to be interacting. Concurrent inhibition of both P/Q type channels and ryanodine receptors generated a significantly larger inhibition that when either channel was inhibited alone (Figure 2C, 2D). We concluded that the P/Q type calcium channels and the ryanodine receptors were independently contributing to the calcium influx signal.

**Figure 3:**
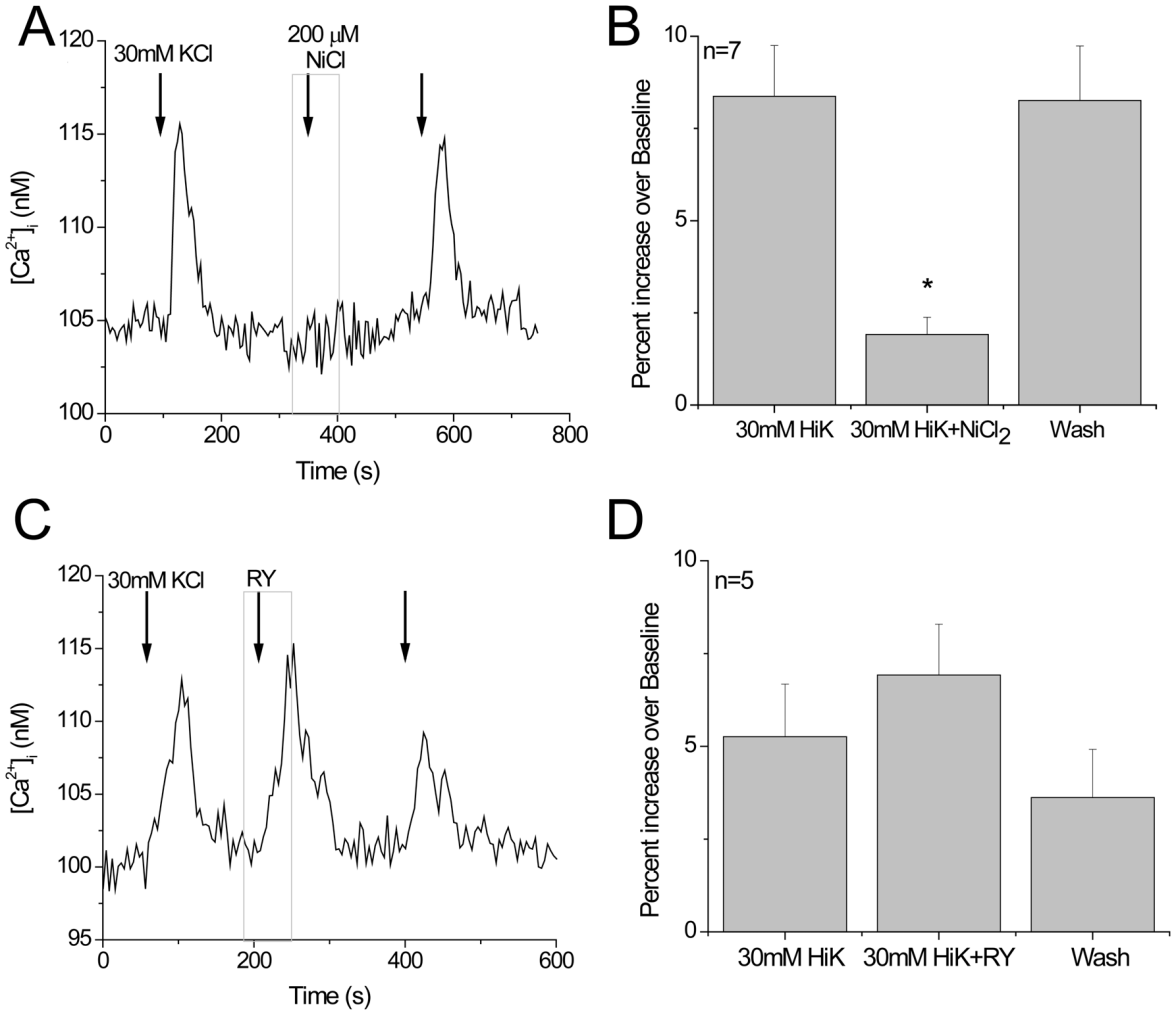
RyRs do not contribute to calcium signal due to T type calcium channels. A) Representative trace showing the inhibition of 30 mM HiK response by NiCl_2_ (200 µM). B) Pooled results showing inhibition of the 30 mM HiK response by NiCl_2_ (200 µM) (n = 7, **p = 0.001). C) Representative trace showing that ryanodine does not affect the calcium influx due to opening T type channels with a small depolarization. D) Pooled results showing no significant inhibition of the 30 mM HiK response by ryanodine (20 µM) (n = 5, p = 0.27).

We also determined if RyRs contribute to calcium influx through T type channels (Ca_V_3.x). To preferentially activate T type calcium channels, we used a lower concentration of HiK (30 mM) to depolarize the cell to approximately −40 mV, which is close to the activation voltage of T type calcium channels [24]. We were able to completely block this calcium-influx response using the T channel blocker NiCl_2_, indicating that this smaller HiK-induced calcium influx was only due to T type calcium channels (Figure 3A, 3B). Results were confirmed with NNC 55-0396 dihydrochloride, and mibefradil, two other T type blockers (data not shown). Application of ryanodine did not affect the calcium current through T type calcium channels and we concluded that RyRs and T type VGCCs do not interact with each other (Figure 3C, 3D).

### Ryanodine Receptors are Associated L type VGCCs

**Figure 4:**
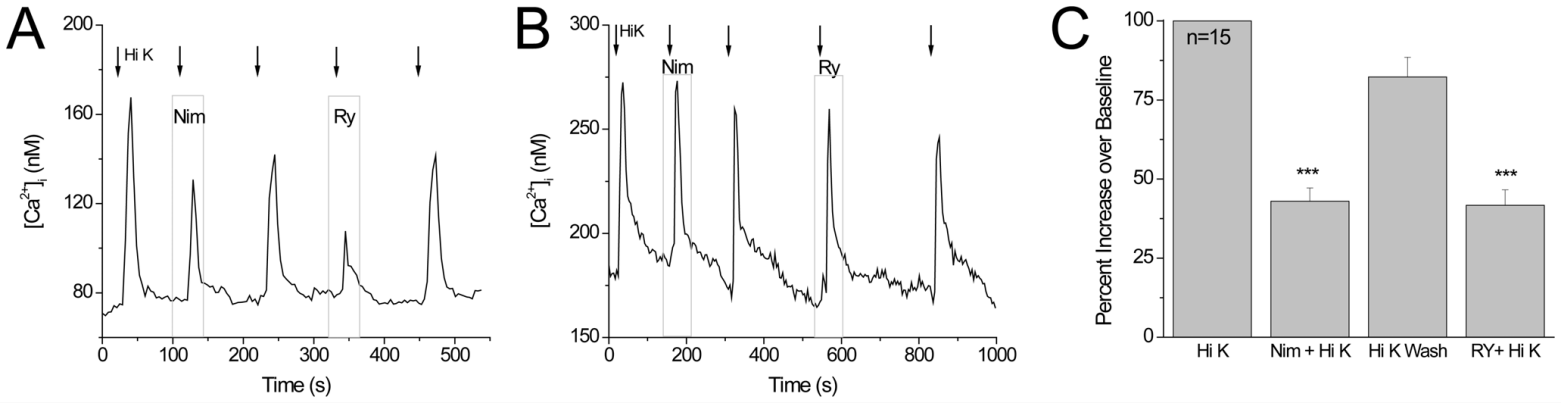
Ryanodine receptors are always found in the Type III taste cells that express L type voltage-gated calcium channels. A) Representative trace showing the inhibition of the calcium influx by nimodipine (10 µM) and ryanodine (20 µM) in the same cell. B) Representative trace showing the lack of RyRs in a cell with no L type channels. C) Pooled results showing the average inhibition by nimodipine and 20 µM ryanodine in the same mouse taste cells (***p<0.001, n = 15).

Since L type calcium channels are expressed in mammalian taste cells [9], [23], [25] and are known to interact with ryanodine receptors in skeletal muscle as well as in the central nervous system [17], [26], [27], we next tested the hypothesis that ryanodine receptors interact with L type calcium channels in taste cells. Nimodipine (10 µM) was used to block L type calcium channels (Ca_V_1.x). Control experiments using two other L type blockers (nifedipine or verapamil) gave comparable results. In the GAD67-GFP expressing taste cells that were tested for both functional RyRs and L type channels, 75% of L type expressing taste cells also expressed RyRs (Figure 4A) and cells that lacked L type calcium channels did not express RyRs (Figure 4B). Follow up experiments used conotoxin MVIIC (1 µM) to block both N and P/Q type calcium channels, nimodipine to block L type calcium channels, and ryanodine to determine if ryanodine receptors were functionally expressed with other high voltage gated-channels in addition to L type channels. We found that multiple voltage-gated channels and ryanodine receptors can all be expressed in the same taste cells but we did not find that ryanodine receptors were ever expressed in Type III taste cells that did not express L type voltage-gated calcium channels (data not shown).

**Figure 5:**
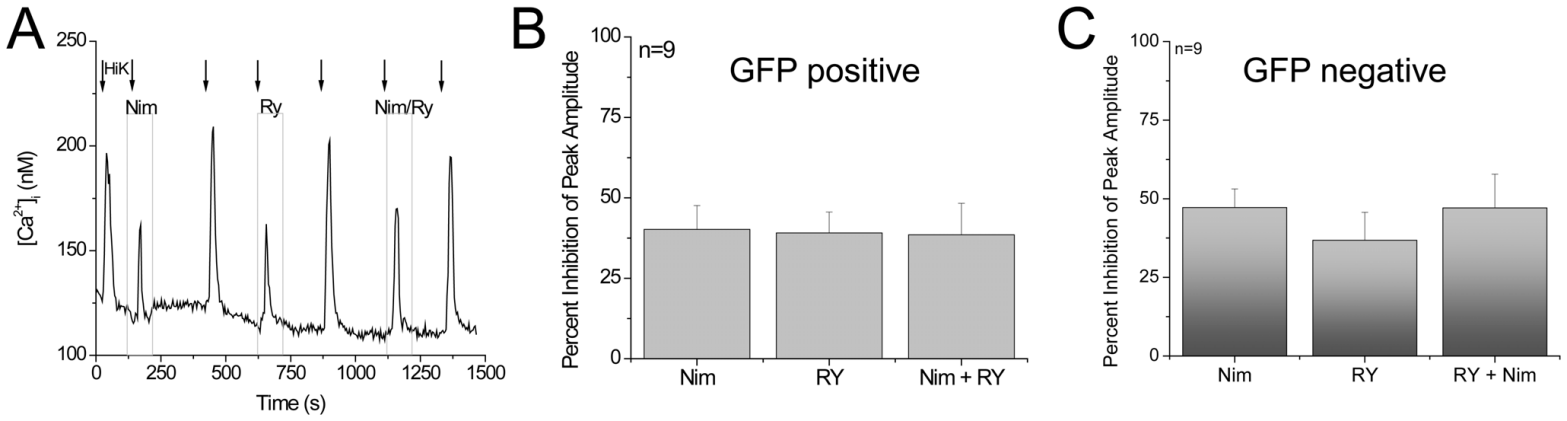
Ryanodine receptors are functionally coupled with L type voltage-gated calcium channels in both GAD-GFP positive and GAD-GFP negative Type III cells. A) Representative trace showing inhibition of the L type calcium channels and ryanodine receptors individually inhibited and then co-inhibited. B) Percent inhibition of the peak amplitude of the evoked calcium influx by nimodipine alone, ryanodine alone and nimodipine+ryanodine together in GAD67-GFP expressing Type III taste cells. No significant differences were detected between the different conditions (n = 7, p = 0.57). C) Percent inhibition of the peak amplitude of the evoked calcium influx by nimodipine alone, ryanodine alone and nimodipine+ryanodine together in the non-GFP expressing Type III taste cells. No significant differences were detected between the different conditions (n = 9, p = 0.62).

Our analysis revealed that separately inhibiting L type calcium channels and ryanodine receptors caused a comparable inhibition in the depolarization induced calcium signal (Figure 4C). These data indicate that ryanodine receptors may be interacting with the L type voltage gated calcium channels. When we measured the inhibition of the calcium influx signal in the presence of nimodipine, ryanodine or nimodipine+ryanodine, we saw no difference in the percent inhibition between these conditions (Figure 5A). We analyzed the peak amplitude of the responses (Figure 5B, n = 7, p = 0.57) to determine the effects of inhibiting the L type channels and the ryanodine receptors on the calcium signal. We found that inhibiting either the L type calcium channels or the ryanodine receptors had comparable effects on the evoked calcium signal in GAD-GFP expressing Type III cells. Inhibiting both channels at the same time also caused approximately the same amount of inhibition. These data suggest that the ryanodine receptor is interacting with the L type channel and is not independently contributing to the calcium signal in the GAD67-GFP expressing taste cells. We repeated these experiments in Type III taste cells that do not express GAD67-GFP and obtained comparable results (Figure 5C, n = 9, p = 0.62). We concluded that despite the presence of multiple voltage-gated channels in taste cells, RyRs preferentially interact with the L type calcium channels in Type III taste cells.

### Nature of Interaction between RyRs and L type Calcium Channels

**Figure 6:**
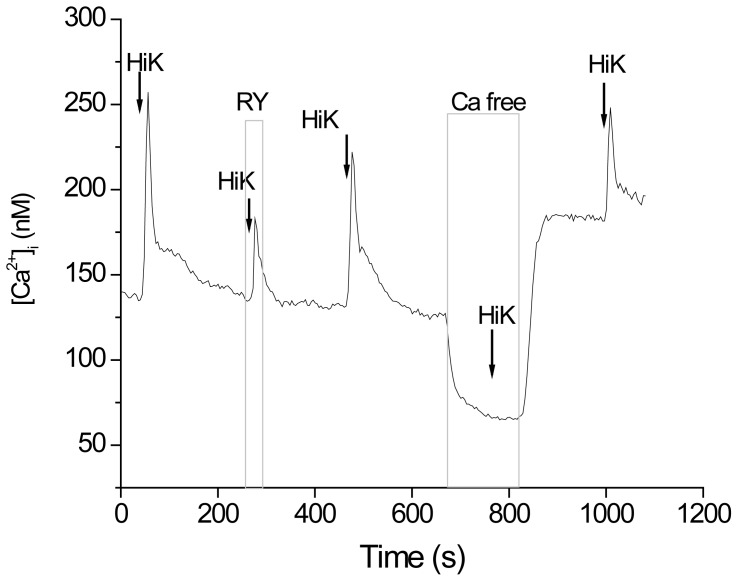
L type voltage-gated calcium channels and RyRs. Representative trace showing the absence of any evoked cytosolic calcium response in an external calcium free solution in a GAD67-GFP taste cell that expresses ryanodine receptors (n = 15).

There are potentially two different ways that L type calcium channels could activate RyRs in taste cells, either by being physically linked to the RyR or through an increase in cytosolic calcium to cause calcium induced calcium release (CICR) through RyRs. To determine how RyRs and L type channels interact in taste cells, we removed external calcium and then depolarized the cell to record any increase in cytosolic calcium. If RyRs and VGCCs are physically coupled, opening VGCCs would open RyRs and calcium would be released from stores. However, if they were not physically coupled, an initial calcium influx from VGCCs would be necessary to activate and open RyRs. We found that in taste cells with ryanodine receptors, no depolarization induced calcium response was ever recorded when the external calcium was absent. Thus, we concluded that there is no physical interaction between the VGCCs and RyRs in taste cells and that RyRs are being activated by elevated cytosolic calcium from the initial calcium influx due to opening L type VGCCs (Figure 6, n = 15).

## Discussion

The goal of this study was to characterize the relationship between RyRs and VGCCs in mammalian taste cells. We previously found that ryanodine receptor 1 is expressed in some Type II and Type III taste cells. Type II taste cells do not express VGCCs but can still express RyRs. In these cells, RyRs contribute to the taste-evoked calcium release signal that depends on the activation of IP_3_ receptors. In Type III taste cells which express VGCCs, the VGCCs and RyR1 interact and the RyRs contribute to the depolarization induced calcium signal in that sub-set of Type III taste cells [13]. However, our earlier study did not determine if RyR1 was interacting with a specific VGCC isoform or if it was activated by any VGCC that was also expressed in the same taste cell. While we found that RyRs can be co-expressed with either N type (Cav2.2) or P/Q type (Cav 2.1) channels in the same Type III taste cells, our data suggest that the RyRs are not associated with these channels. Instead, we found that there is specificity in the VGCC isoform that interacts with RyR1; in particular RyRs appear to only interact with L type calcium channels in taste cells. Interestingly, an earlier study did not find that 800 nM ω-conotoxin GVIA which blocks N type VGCCs had an effect on the depolarization evoked calcium influx [25], however, we found robust inhibition of the depolarization-evoked calcium influx by GVIA in some taste cells. We did not find that ω-conotoxin GVIA inhibition in all Type III taste cells, so it is possible the earlier study did not analyze Type III taste cells that expressed N type VGCCs.

In skeletal muscles, RyR1 and L type VGCCs physically couple to each other to facilitate excitation-contraction coupling [28]. There is also evidence of a physical interaction between RyRs and L type VGCCs in multiple types of neurons including hypothalamic magnocellular neurons, dorsal column axons and hippocampal pyramidal neurons [14]–[17], [29]. While in skeletal muscle the relationship between L type calcium channels and RyR has a well-established role in muscle contraction [28], the role of this voltage-induced calcium release (VICaR) through the coupling of L type channels and RyRs has not been clearly defined in neurons. In hypothalamic neurons, VICaR has two effects on cytosolic calcium levels: 1) an increase in the number of quantal size calcium syntillas, or focal calcium transients, in the synaptic terminals and 2) an increase in global cytosolic calcium levels. Calcium syntillas depend on L type channels coupling with RyR1 and occurred when membrane potentials increase from −80 mV to −60 mV. Over this range of membrane potential, there is no voltage-gated calcium influx but the frequency of these calcium transients significantly increases, specifically through the activation of RyR1 [14], [15], [29]. Currently, the physiological role of these calcium syntillas is not well defined and seems to vary with cell type. Calcium syntillas do not cause exocytosis in magnocellular neurons [30] or in chromaffin cells [31] but make significant contributions to initiating exocytosis in dorsal root ganglion neurons [32]. In chromaffin cells, the syntilla calcium is released into a microdomain that is different from the site of exocytosis [31] where it appears to suppress exocytosis [33]. Thus, in multiple neurons, RyRs physically couple to L type calcium channels to generate small transient calcium syntillas and contribute to depolarization evoked global calcium increases. While the functions of these events vary, they each make important contributions in regulating neurotransmitter release.

RyRs and L type VGCCs are also important in contributing to contraction in cardiac muscles, but in these cells, there is not a physical coupling between the RyRs and L type channels. Instead, a tetrad of RyRs surrounds each L type VGCC [34] and the depolarization-induced calcium influx from the L type calcium channel activates the RyRs causing a highly amplified localized calcium signal that activates nearby clusters of RyRs. This signaling event often initiates and propagates a calcium wave in these cells via CICR by RyRs. CICR is a ubiquitous positive feedback process that ensures the needed sensitivity and magnitude of calcium signal. CICR occurs in very diverse physiological processes ranging from muscle contraction to neurotransmitter release [35]–[37]. CICR can function to provide a high gain amplification of the initial calcium signal and in the case of calcium waves, cause a localized signal to be broadcast over the entire cytoplasm [38]. Yet more recent studies have revealed that CICR is often tightly correlated to the magnitude of the trigger calcium signal, which is primarily calcium influx through calcium channels [39], [40]. In dorsal root ganglion neurons, CICR was shown to be comprised of multiple components: CICR that contributes to a cytosolic calcium increase across the entire cell and another type of CICR that occurs at subsurface cisternae areas in which the CICR is tightly controlled by the localized calcium influx in that area. In these focused areas of CICR at the plasma membrane, the calcium increases regulate both membrane limited calcium dependent events as well as trigger calcium increases that contribute to global calcium changes. These localized CICR events appear to be self-regulating and do not require termination mechanisms other than the spontaneous closing of the ryanodine channels [41]. In dorsal root ganglion neurons, these localized CICR events are responsible for 60% of the calcium dependent exocytosis [32]. Clearly, the relationship between RyRs and calcium influx through VGCCs are critical for normal vesicular release in multiple types of neurons.

In this study, we showed that RyR1 is selectively associated with L type calcium channels in mammalian taste cells, but our initial experiments did not determine if RyR1 and L type were physically connected or just functionally associated. Earlier studies that have shown a physical coupling between RyR and L type channels were able to demonstrate either physiologically or biochemically that these two proteins can interact in neurons just as they do in muscle. Immunoprecipitation in rat brain membrane preparations demonstrated a physical linking between RyR and L type calcium channels but this study did not demonstrate a functional association [26]. Physiological studies have demonstrated that RyR and L type channels can be physically linked in neurons using calcium imaging. In these studies, even in the absence of external calcium, cytosolic calcium levels increased in response to membrane depolarization. These increases were blocked by L type channel antagonists or high levels of ryanodine, which prevent RyRs from opening [15], [17], [32]. We did not perform immunoprecipitation to determine the potential nature of the interaction between RyR and L type channels since this experiment would only determine a biochemical but not a physiological interaction. Instead, we used calcium imaging in taste cells to determine the effect of removing external calcium on the subsequent depolarization induced calcium elevations (Figure 6).

We found that in the absence of external calcium, there was never an increase in cytosolic calcium, even though RyRs were functionally expressed in those cells. Our data suggest that the CICR in taste cells depends on a close apposition but not a physical association between the two proteins and that calcium influx through the L type VGCCs activates nearby RyRs on the endoplasmic reticulum (ER). Therefore the calcium influx response in the taste cells in which RyRs and VGCCs functionally interact will have a ryanodine insensitive and a ryanodine sensitive component. The ryanodine insensitive component represents the trigger, which is the initial calcium influx via VGCCs, while the ryanodine sensitive component involves the calcium release from stores due to CICR. This mechanism is likely an important amplifier of intracellular calcium signals, being capable of producing large amplitude calcium transients in taste cells. Since L type calcium channels are sensitive to a calcium dependent inactivation, coupling their activity to CICR by RyRs may ensure that a sufficient calcium signal is generated in response to cell depolarization.

A likely role for CICR in taste cells is synaptic transmission and there is evidence that CICR in other sensory neurons contributes to neurotransmitter release. In salamander and mouse retina, CICR boosts synaptic release from rod photoreceptor terminals by amplifying the effects of calcium entering through L type VGCCs [42]–[44]. The inner hair cell synapse of the mammalian cochlea also shows CICR involvement in synaptic transmission [45]. It is therefore possible that that CICR also enhances synaptic release in mouse taste cells, however further experimentation is required to elucidate the exact mechanism involved in this process. It has been reported that a subset of Type III taste cells lack GAD67-GFP expression and express L type Ca_V_1.2 [25]. Another study using RT-PCR analysis, reported both L type Ca_V_1.2 and Ca_V_1.3 expression in mammalian taste cells [9]. Based on these earlier studies and our data, we predict that the predominant L type isoform that functionally interacts with the RyR in the GAD67-GFP taste cells is the L type Ca_V_1.3. While direct measurements are needed to confirm this hypothesis, Ca_V_1.3 has been shown to associate with different RyR isoforms in the CNS [26], [27]. The present study shows that L type VGCCs functionally, but not physically, interact with RyRs to enhance the calcium influx signal. This interaction is likely important for controlling and fine-tuning the calcium signals, which are vital elements of taste signal processing.
